# Limbic Responses to Aversive Visual Stimuli during the Acute and Recovery Phase of Takotsubo Syndrome

**DOI:** 10.3390/jcm11164891

**Published:** 2022-08-20

**Authors:** Ruth Steiger, Noora Tuovinen, Agne Adukauskaite, Thomas Senoner, Philipp Spitaler, Valentin Bilgeri, Agnieszka Dabkowska-Mika, Christian Siedentopf, Axel Bauer, Elke Ruth Gizewski, Alex Hofer, Fabian Barbieri, Wolfgang Dichtl

**Affiliations:** 1 University Hospital for Neuroradiology, Medical University of Innsbruck, 6020 Innsbruck, Austria; 2 Neuroimaging Research Core Facility, Medical University of Innsbruck, 6020 Innsbruck, Austria; 3 Division of Psychiatry I, University Hospital for Psychiatry, Psychotherapy, Psychosomatics and Medical Psychology, Medical University of Innsbruck, 6020 Innsbruck, Austria; 4 University Hospital for Internal Medicine III (Cardiology and Angiology), Medical University of Innsbruck, 6020 Innsbruck, Austria; 5 University Hospital for Anesthesiology, Medical University of Innsbruck, 6020 Innsbruck, Austria; 6 University Hospital for Cardiology, Charité—Campus Benjamin Franklin, 12203 Berlin, Germany

**Keywords:** brain–heart axis, limbic system, insular cortex, takotsubo syndrome, myocardial infarction, task-based fMRI

## Abstract

The role of the limbic system in the acute phase and during the recovery of takotsubo syndrome needs further clarification. In this longitudinal study, anatomical and task-based functional magnetic resonance imaging of the brain was performed during an emotional picture paradigm in 19 postmenopausal female takotsubo syndrome patients in the acute and recovery phases in comparison to sex- and aged-matched 15 healthy controls and 15 patients presenting with myocardial infarction. Statistical analyses were performed based on the general linear model where aversive and positive picture conditions were included in order to reveal group differences during encoding of aversive versus positive pictures and longitudinal changes. In the acute phase, takotsubo syndrome patients showed a lower response in regions involved in affective and cognitive emotional processes (e.g., insula, thalamus, frontal cortex, inferior frontal gyrus) while viewing aversive versus positive pictures compared to healthy controls and patients presenting with myocardial infarction. In the recovery phase, the response in these brain regions normalized in takotsubo syndrome patients to the level of healthy controls, whereas patients 8–12 weeks after myocardial infarction showed lower responses in the limbic regions (mainly in the insula, frontal regions, thalamus, and inferior frontal gyrus) compared to healthy controls and takotsubo syndrome patients. In conclusion, compared to healthy controls and patients suffering from acute myocardial infarction, limbic responses to aversive visual stimuli are attenuated during the acute phase of takotsubo syndrome, recovering within three months. Reduced functional brain responses in the recovery phase after a myocardial infarction need further investigation.

## 1. Introduction

Takotsubo syndrome (TTS) is characterized by sudden onset left ventricular dysfunction typically occurring in the aftermath of psychological and/or physical stress. Its incidence has further increased during the ongoing pandemic [1]. Although originally believed to be a rather benign disease, this neuro-cardiac syndrome has a long-term clinical outcome comparable with acute myocardial infarction (MI).

The recently supposed neuro-cardiac basis of TTS is supported by magnetic resonance imaging (MRI) studies show distinctive differences in brain grey matter content, task-based functional MRI (fMRI) responses as well as brain functional and structural connectivity compared to healthy controls [2,3,4,5,6,7,8]. 18F-flourodeoxyglucose positron emission tomography/computed tomography (18F-FDG-PET/CT) studies have suggested that differences in the limbic system may be present already before or with the onset of a TTS episode [9,10]. Similarly, an IAPS (International Affective Picture System) fMRI-study showed that distributed brain activity patterns of the limbic system could comprise affective neural correlates of pre-clinical atherosclerosis [11].

These structural and functional brain abnormalities in TTS are often lateralized and mainly involve the limbic system (hippocampus, amygdala, thalamus, cingulate gyrus, insula). These areas are responsible for fine-tuning emotions, such as anxiety, anger, and stress, as well as the cardiac autonomic balance. In the chronic phase of TTS, previous resting-state and task-based fMRI studies found both a stronger connectivity between the midcingulate cortex and the parasympathetic primary motor area, which are brain areas involved in pain experience regulation and visceromotor control (in comparison to patients with previous acute MI) [12], as well as lower response of limbic regions during emotional picture paradigm (in comparison to healthy age- and gender-matched control subjects) [13], respectively. 

In contrast to accumulating cross-sectional evidence on structural and functional brain differences in the chronic phase of TTS compared to controls, neuroimaging data obtained in the acute phase (and compared in the same cohort during short-term follow-up) are scarce. In particular, there is a knowledge gap regarding how the limbic system responds to emotional stimuli in the acute phase of TTS and how this alters to the early recovery phase.

The aim of the herein presented TAKINSULA study was therefore to investigate emotional responses during task-based fMRI of the brain in the acute phase of a TTS episode with a longitudinal assessment after 8–12 weeks, in comparison to sex- and aged-matched healthy controls (HC) and patients presenting with MI. As TTS is often triggered by a stressful event, limbic responses to affective pictures might differ in TTS patients as compared to patients suffering from acute MI, as well as compared to HC.

## 2. Patients and Methods

Between 2014 and 2019, postmenopausal women were recruited at the Department of Internal Medicine III, Medical University of Innsbruck, Austria. Baseline characteristics are shown in Table 1. The diagnosis of TTS was performed by invasive contrast left ventriculography in the setting of emergency coronary angiography. Cardiac MRI was later performed in selected cases to confirm diagnosis where the presentation was not entirely sure to be TTS. Exclusion criteria were any practical reason prohibiting the performance of a brain MRI within the following 72 h, limited capability to communicate in German, implantation of a cardiac electronic device or a mechanical heart valve, and general contraindications against performing an MRI examination (claustrophobia, severe obesity). None of the TTS patients had a previous diagnosis of depression or anxiety-related disorders.

Brain MRI in TTS and MI patients was performed in the acute phase on the second or third day after hospital admission in a clinically stable situation free of pulmonary congestion and/or sustained new-onset arrhythmias. HC did not suffer from overt neurological or psychiatric diseases or any relevant medical conditions except for antihypertensive and/or lipid-lowering drugs. Brain MRIs in TTS and MI patients were repeated in the recovery phase after 8–12 weeks.

The study procedures were performed according to the Declaration of Helsinki and approved by the ethics committee of the Medical University of Innsbruck (approval code 20140308-928). All subjects signed informed consent form prior to inclusion in the study. The study was registered at ClinicalTrials.gov (NCT02240056).

Descriptive statistics were computed with IBM SPSS Statistics software version 26.0 Gaussian distribution was confirmed by the Shapiro–Wilk test (α < 0.05), histograms, and QQ plots in participants. The group differences of normally distributed data were analyzed by parametric tests (unpaired *t*-test, 2-tailed). Non-Gaussian distributed variables were assessed by Mann–Whitney U-test (α < 0.05).

MRI acquisition: Anatomical and functional sequences were obtained using a 3 Tesla MRI scanner (Magnetom Verio Syngo, Siemens Erlangen). For the anatomical measurement, a T1-weighted magnetization prepared rapid acquisition gradient echo (MPRAGE) sequence with following parameters was acquired: repetition time (TR) = 1950 ms, echo time (TE) = 3.3 ms, flip angle = 9°, field of view (FOV) = 220 × 178.75 mm^2^, in-plane resolution = 0.9 × 0.7 mm, slice thickness = 1 mm, and gap = 0.5 mm. T2-weighted turbo inversion recovery magnitude (TIRM) sequence parameters were set to: TR = 6150 ms, TE= 97 ms, flip angle = 150°, and FOV = 220 × 171.88 mm^2^. Participants were laid head-first supine in the scanner with foam pads to stabilize the head and wore earplugs to protect them from the scanner noise. For task-based fMRI acquisition, a GRAPPA EPI (GeneRalized Autocalibrating Partially Parallel Acquisitions echo-planar-imaging) sequence was employed with following parameters: TR = 2400 ms, TE = 30 ms, flip angle = 90°, matrix = 96 × 96, FOV = 220 mm, slice number = 37, slice thickness = 2.5 mm, distance factor = 40%, accelerator factor = 2, and in-plane resolution = 2.3 × 2.3 mm. For each run, 175 functional volumes were acquired with acquisition time = 7:09 min.

**Figure 1:**
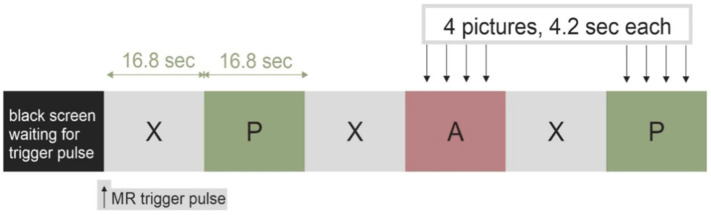
The task-based functional magnetic resonance imaging (fMRI) visual paradigm used in the TAKINSULA study, which presented 24 positive (P) and 24 aversive (A) pictures from the International Affective Picture System (IAPS) in a block design together with a fixation cross (X).

fMRI paradigm design and stimuli: Task-based fMRI acquisition, which assesses emotional picture processing, was applied, during which TTS and MI patients as well as HC passively watched positive (non-aversive) and aversive pictures selected from the International Affective Picture System (IAPS) [14]. The pictures were selected for their affective valence and arousal ratings, where positive values elicited diverse positive emotions, whereas aversive emotions (such as disgust or fear) rated highly negative [15]. The picture stimuli were displayed via video goggles (NordicNeuroLab, Norway), mounted directly at the 12-channel head coil, and were presented in a block design (block length = 16.8 s) with nordicAktiva software (NordicNeuroLab). Each presentation block (see Figure 1) was preceded by a fixation cross, followed by a display of 4 pictures in a series, either positive or aversive (6 blocks each). Blocks were randomly shuffled in order to avoid biasing due to anticipation. During the paradigm, 24 positive and 24 aversive pictures were shown. The pictures were distributed randomly across the blocks.

Data analyses: The anatomical brain scans were controlled for lesions (e.g., insular, limbic) by an experienced neuroradiologist (ERG). All data were processed using SPM12 (Wellcome Department of Cognitive Neurology, London, UK) based on Matlab R2018a. Functional images of each run (two appointments for each patient) were motion corrected, realigned and normalized to the EPI Montreal Neurological Institute (MNI) template space using the deformation field parameters and smoothed using an 8 mm full width at half maximum (FWHM) Gaussian kernel. Statistical analyses were performed based on the general linear model where two conditions of interest, e.g., aversive and positive, based on the onsets of different picture conditions, were included. The BOLD response for the pictures was modeled using the canonical form of the hemodynamic response function. Its duration was set to the time when the pictures were presented: 4.2 s per picture, 16.8 s per block. A high-pass filter (cut-off frequency: 1/120 Hz) was used to remove low frequency drifts. The six motion parameters from the realignment procedure per block were embedded as parameters of no interest into the model. A contrast ‘aversive vs. positive’ was investigated in order to reveal group differences (i.e., TTS _acute phase_ vs. HC; TTS _recovery phase_ vs. HC; TTS _acute phase_ vs. TTS _recovery phase_; MI _acute phase_ vs. HC; MI _recovery phase_ vs. HC; TTS _acute phase_ vs. MI _acute phase_; TTS _recovery phase_ vs. MI _recovery phase_; MI _acute phase_ vs. MI _recovery phase_) in the brain responses.

First level analyses were applied for the single groups and second level analyses for group comparisons were based on the contrasts from the first level-analysis. For all analyses, clusters that survived an initial uncorrected *p*-value of less than 0.001 and, additionally, family wise error (FWE) corrected with a *p*-value of less than 0.05 were considered.

## 3. Results

### 3.1. Clinical Characteristics

The baseline demographics of the 49 participants recruited in the study are shown in Table 1. No obvious brain lesions were visible in the routine MR sequences.

### 3.2. Group Comparisons

fMRI analyses investigated group (i.e., TTS, MI, HC) differences in the BOLD responses during the IAPS picture paradigm, during which aversive and positive pictures were contrasted (see Figure 1). The group analyses were applied with an uncorrected *p* value < 0.001. However, further group comparisons with FEW statistics led to no surviving voxels.

**Figure 2:**
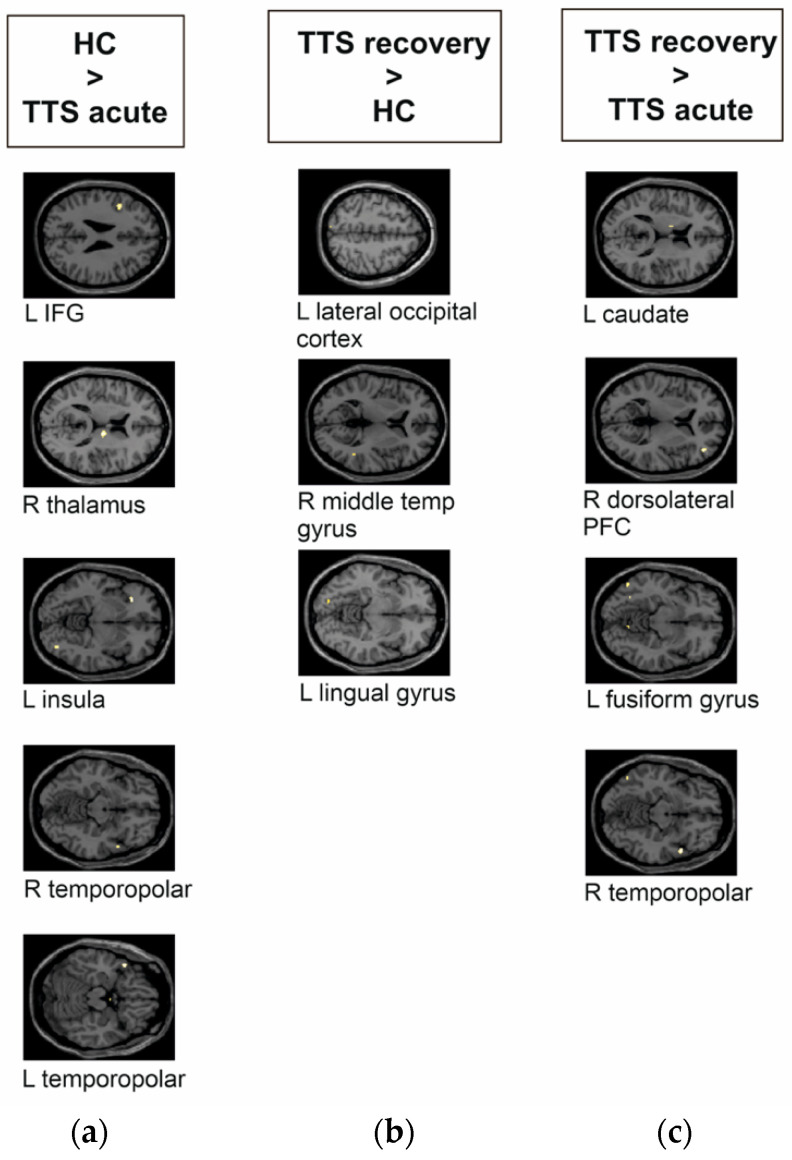
Group differences of takotsubo syndrome patients (TTS) and healthy controls (HC) during the emotional task-based fMRI paradigm during the decoding of aversive versus positive pictures from the IAPS. The brain images are presented in MNI space. (**a**) TTS _acute phase_ compared to HC had significantly lower mean activity in the left inferior frontal gyrus (IFG), right thalamus, left insula, and in the right and left temporopolar areas. (**b**) TTS _recovery phase_ compared to HC had significantly higher activity within the left lateral occipital cortex, right middle temporal gyrus, and left lingual gyrus. (**c**) TTS _acute phase_ compared to TTS _recovery phase_ had significantly lower responses in the left caudate, right dorsolateral prefrontal cortex (PFC), left fusiform gyrus, and right temporopolar area.

Analyses revealed group differences in decoding aversive versus positive pictures, where TTS _acute phase_ showed significantly lower mean activity in the left insula, the right thalamus, bilateral temporopolar areas, and the left inferior frontal gyrus (IFG) compared to HC (see Figure 2a and Table 2). TTS _recovery phase_ presented significantly higher activity with small clusters (<10 voxels) within the left lingual gyrus, the left lateral occipital cortex, and in the right middle temporal gyrus compared to HC (see Figure 2b). Significantly higher responses in the TTS recovery phase were noted in the right dorsolateral prefrontal cortex (PFC) and the right temporopolar area, along with small clusters within the left fusiform gyrus and the left caudate compared to the TTS _acute_ _phase_ (see Figure 2c).

**Figure 3:**
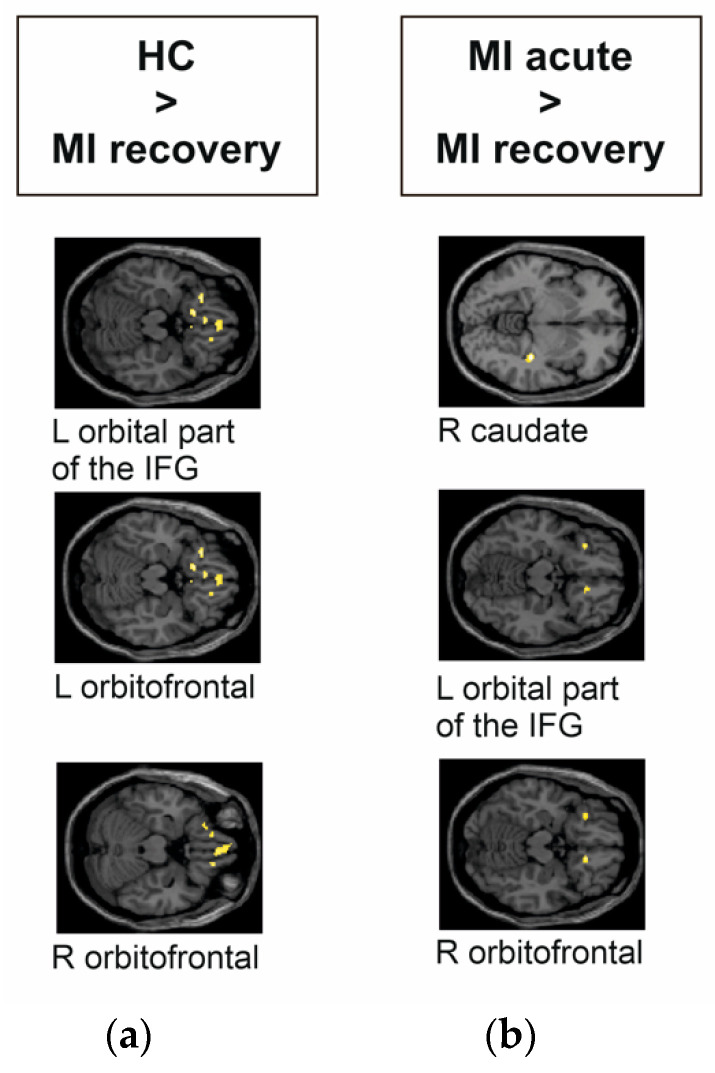
Group differences of myocardial infarction (MI) patients and HC during the emotional task-based fMRI paradigm during the decoding of aversive versus positive pictures from the IAPS. The images were presented in MNI space. MI _acute phase_ compared to HC showed no significant differences. (**a**) MI _recovery phase_ compared to HC had significantly lower responses in the left orbital part of the IFG, and in the left and right orbitofrontal regions. (**b**) MI _acute phase_ compared to MI _recovery phase_ had significantly higher activity in the right caudate, left orbital part of the IFG, and right orbitofrontal region.

In the acute phase, MI showed no significant group differences compared to HC during the presentation of visual stimuli. In contrast, HC had significantly higher activity in the left and right orbitofrontal region and the left orbital part of the IFG compared to MI _recovery phase_ (see Figure 3a and Table 3). MI _acute phase_ had significantly higher activity in the right orbitofrontal, the left orbital part of the IFG, and the right caudate compared to MI _recovery_ _phase_ (see Figure 3b).

**Figure 4:**
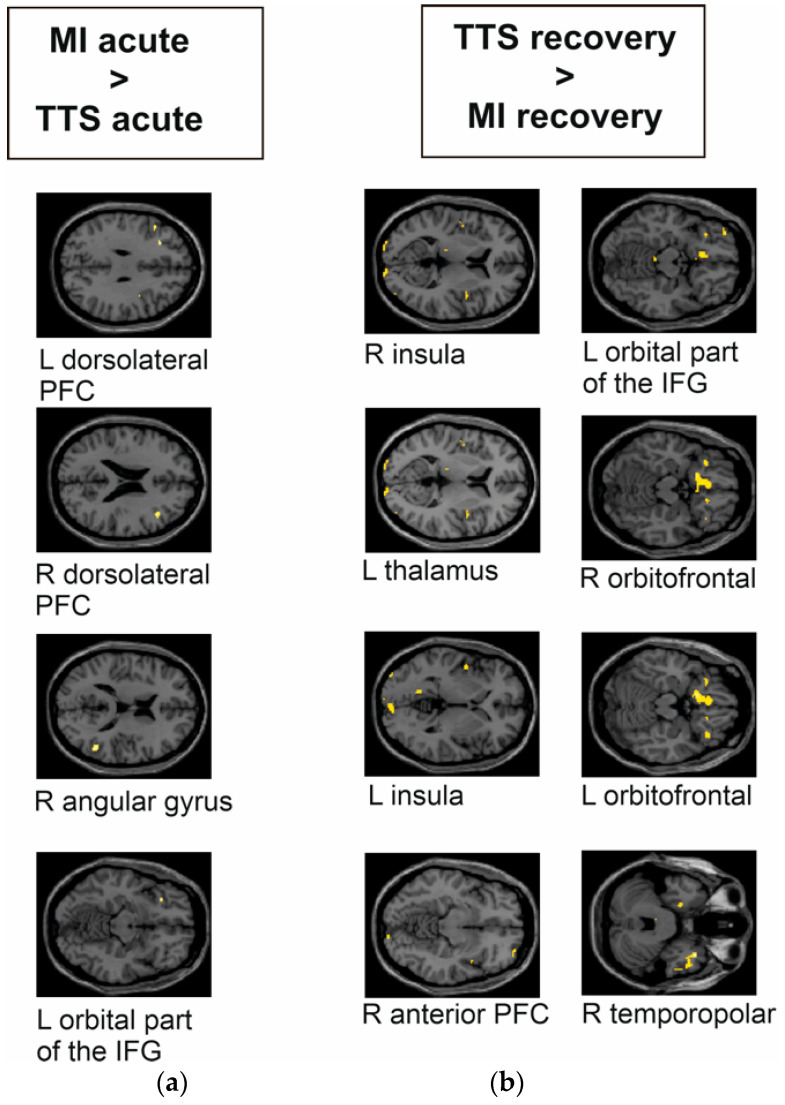
Group differences of TTS and MI during the emotional task-based fMRI paradigm during the decoding of aversive versus positive pictures from the IAPS. The images are presented in MNI space. (**a**) TTS _acute phase_ compared to MI _acute phase_ had significantly lower response in the left and right dorsolateral PFC, right angular gyrus, and left orbital part of the IFG. (**b**) TTS _recovery phase_ compared to MI _recovery phase_ had significantly higher response bilaterally in the (**right**) insula, left thalamus, (**left**) insula, (**right**) anterior PFC, (**left**) orbital part of the IFG, (**right**, **left**) orbitofrontal area, and (**right**) temporopolar area.

In the acute phase, MI patients had significantly higher activity bilaterally in the dorsolateral PFC, the right angular gyrus, and the left orbital part of the IFG compared to TTS patients (see Figure 4a and Table 4). In the recovery phase, TTS patients had significantly higher activity bilaterally in the insula, the right anterior PFC, the right and left orbitofrontal area, the left orbital part of the IFG, and in small clusters in the right temporopolar area and the left thalamus compared to MI patients (see Figure 4b).

## 4. Discussion

While around seven percent of TTS events are associated with clear neurologic triggers, such as seizures or intracranial bleeding [16], there is evolving evidence that the brain–heart axis is genuinely involved in the general pathogenesis of this condition, mainly involving the central autonomic nervous system (for review, see [17,18]). As no current TTS therapies exist, this underexplored field of neuro-cardiology might be of particular interest to develop new management approaches, including cognitive behavioral therapy and/or physical exercise training. From a broader perspective, brain abnormalities may play a role in chronic heart failure, as this patient group was recently reported with a markedly reduced hippocampal brain volume associated with impaired cognitive function in the COGNITION.MATTERS-HF trial [19].

The TAKINSULA study was set out to investigate emotional responses during task-based fMRI in the acute and recovery phase (after 8–12 weeks) of a TTS episode. HC and patients with MI were included as control groups. In comparison to HC, TTS patients had a lower activity in the left insula, the right thalamus, the bilateral temporopolar areas, and the left IFG during the acute phase of the event. In TTS patients lower brain responses were found upon comparison to patients suffering from MI in the acute phase, namely in the left and right dorsolateral PFC, the right angular gyrus, and the left orbital part of the IFG.

In contrast to these lower brain responses during the acute phase of TTS, patients in the recovery phase after the TTS event generally showed higher brain responses upon aversive versus positive visual stimuli: (a) within the left visual cortex (i.e., lingual gyrus and lateral occipital cortex) and the right middle temporal gyrus as compared to HC; (b) in the bilateral insula, the right anterior PFC, the left and right orbitofrontal area, left orbital part of the IFG, right temporopolar area, and the left thalamus as compared to the recovery phase after a MI; and (c) in the right dorsolateral PFC, the left fusiform gyrus, the left caudate, and the right temporopolar area as compared to the responses during the acute phase of the TTS episode.

Patients presenting with MI reacted the opposite way: no differences were found during the acute phase of MI as compared to HC, but brain responses attenuated in the recovery phase, with lower activity in the left and right orbitofrontal regions and the left orbital part of the IFG as compared to HC. This held true in the longitudinal analysis of patients with MI as well. As compared to the recovery phase, patients during the acute phase of MI showed higher responses in the right orbitofrontal area, left orbital part of the IFG, and the right caudate.

The noted differences in the insular cortex, the thalamus, the frontal cortex, and the angular gyrus are in line with recent findings given their well-known roles in affective and cognitive processes. In particular, lower volume and cortical thickness [2,6], lower functional connectivity [2,3] and activation differences [5,7] are found in the insula of TTS patients, and sudden emotional stress together with insular abnormalities were linked to QTc interval prolongation [20]. In addition, strokes involving the left insular region are associated with adverse cardiovascular outcomes [21].

Our results are further supported by previous TTS that noted lower thalamic volume [2] and, in general, the lower functional connectivity of PFC [8] and the limbic system [6]. The frontal cortex, involving the dorsolateral PFC and the IFG, were previously noted with a lower involvement during IAPS task-based fMRI in decoding negative versus neutral pictures and lower activity in the bilateral superior parietal lobe when processing negative versus positive expected pictures in the recovery phase of TTS compared to HC [13]. Our study showed that TTS _recovery phase_ compared to HC had significantly higher mean activity within the left lateral occipital cortex, right middle temporal gyrus, and left lingual gyrus during the decoding of negative versus positive pictures. The different follow-up timeline (average 27 months vs. average 2.5 months) may however explain some of the noted differences.

In addition, the IFG had higher functional connectivity as a part of a network during rest in TTS patients compared to HC [5]. All these studies consistently point to an involvement of limbic brain regions in TTS functionally and structurally.

Such changes in regions associated with emotional processing may explain why stressful life events can trigger TTS episodes as these regions are co-involved in the regulation of the cardiovascular system. In fact, this study shows differences in activity in limbic regions during emotional processing already in the acute phase of TTS compared to HC and MI. Such volumetric and functional connectivity differences in the acute phase have been previously demonstrated in a partly overlapping patient group [2]. Therefore, the presented results suggest that such differences are not a consequence of TTS but are already present in early stages. ^18^F-FDG-PET/CT studies have suggested that differences in the limbic system may be present already before or with the onset of a TTS episode [9,10].

The noted differences and longitudinal changes in temporal regions (i.e., bilateral temporopolar areas, temporal pole) are in line with previous studies, which noted that the temporal pole has lower structural connectivity [6], differences in functional connectivity during resting-state fMRI [2,3,5], and lower activity during task-based fMRI [5] in TTS compared to HC. In fact, the temporal pole is a paralimbic region interconnected with both the amygdala and the orbital frontal cortex and is therefore involved in socio-emotional processing (for review, see [22]).

The changes in the response to emotional stimuli from acute to the recovery phase of TTS noted in the dorsolateral PFC, the fusiform gyrus, the caudate, and the temporopolar areas may be related to the role of these regions in different aspects of emotional processing. The fusiform gyrus has a role in facial recognition and perception [23], the caudate is considered as an interface for emotional and cognitive processes [24], and the emotional role of the PFC and temporopolar areas was presented earlier. It appears that the response to socio-emotional content especially varies between the acute and recovery phase as displayed by the activation variations in the fusiform gyrus and temporopolar area.

The major limitation of the TAKINSULA study is its relatively small sample size. Nevertheless, the demographic characteristics of the different groups were comparable. In addition, the study used a block design to increase its statistical power in order to detect exiguous differences between the groups, which may be pursued more sensitively with a ROI-based approach. However, ROI-based approaches may suffer from the selection bias of regions with expected changes. Finally, as there were no acquired MRI data prior to the onset of TTS, it cannot be determined whether the limbic responses were the cause or the consequence of the TTS event. However, a previous study [9] provides support for metabolic brain changes before the onset of TTS, and pre-existing psychiatric disorders with known changes in limbic brain regions have been suggested as risk factors for TTS [25,26]. 

## 5. Conclusions

**Figure 5:**
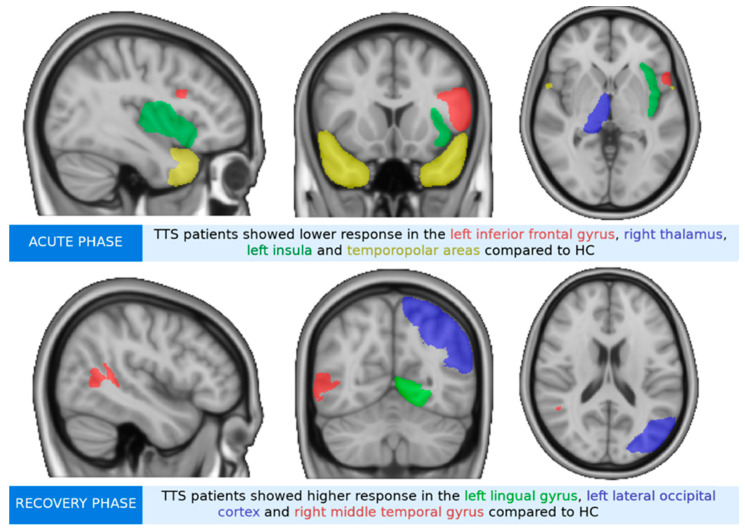
Graphic abstract of the main findings.

The limbic system of TTS patients responds differently to aversive versus positive emotional pictures in the acute phase of the event compared to HC during task-based fMRI. However, these limbic responses of TTS patients recover within 8–12 weeks suggesting that these effects are only transient. An improvement above the levels of HC could be a sign of a temporary compensation mechanism to retrieve the necessary brain functions after short-term recovery (see Figure 5).

In contrast, patients suffering from myocardial infarction appear to develop lower responses to emotional stimuli within time. This unexpected finding needs further investigations and could be a sign of long-term effects on the brain function primarily caused by cardiac damage.

## Figures and Tables

**Table 1:** Sample characteristics of HC, as well as TTS and MI patients at baseline. Distributions of demographic and clinical data for participants are presented as means ± standard deviation or median ± interquartile range. TTS: takotsubo syndrome, MI: myocardial infarction, HC: healthy controls.

	TTS (N = 19)	MI (N = 15)	HC (N = 15)	P (TTS vs. MI/TTS vs. HC)
Age, years	68.3 ± 8.1	64.9 ± 7.2	62.5 ± 10.2	0.037/0.11
Heart rate, beats/min	80.5 ± 12.4	69.8 ± 12.7	66.9 ± 7.2	0.009/<0.001
Body mass index	25.3 ± 4.0	25.3 ± 5.3	24.6 ± 2.7	0.484/0.285
Serum creatinine, mg/dL	0.77 ± 0.12	0.82 ± 0.15	0.95 ± 0.29	0.16/0.0108
Troponin T, ng/L (peak)	512.9 ± 472.8	4177.2 ± 4721.1	6.9 ± 2.3	0.001/<0.001
NT-proBNP, ng/L (peak)	3018 ± 3053	2049 ± 4040	195 ± 137	0.235/<0.001
LVEF, %	50.3 ± 14.8	49.9 ± 11.7	58.4 ± 4.4	0.475/0.026
Medical history				
Arterial hypertension	12 (63.2)	10 (66.6)	9 (60.0)	0.419/0.428
Diabetes mellitus	2 (10.5)	0 (0)	0 (0)	-
Hyperlipidemia	11 (57.9)	11 (73.3)	8 (53.3)	0.182/0.399
COPD	1 (5.3)	0 (0)	0 (0)	-
Peripheral artery disease	1 (5.3)	0 (0)	0 (0)	-

**Table 2:** Significant group differences between HC and TTS while viewing aversive versus positive pictures during emotional task-based fMRI paradigm.

Group Contrast	Number of Voxels	MNI Coordinate	Region	T Value	*p* Value
TTS acute phase < HC	40	−42, 12, 26	L IFG	3.86	0.000
	39	14, −12, 14	R thalamus	4.21	0.000
	26	−28, 28, −6	L insula	4.15	0.000
	13	−46, 18, −18	L temporopolar	4.19	0.000
	6	56, 10, −12	R temporopolar	3.62	0.001
TTS recovery phase > HC	5	−16, −84, −8	L lingual gyrus	3.67	0.001
	5	−8, −80, 52	L lateral occipital cortex	3.55	0.001
	2	46, −46, 6	R middle temporal gyrus	3.59	0.001
TTS recovery phase > TTS acute phase	25	42, 44, 6	R dorsolateral PFC	4.24	0.000
	19	56, 10, −12	R temporopolar	4.14	0.000
	9	−12, −4, 14	L caudate	3.65	0.001
	8	−52, −66, −10	L fusiform gyrus	3.67	0.001

IFG: inferior frontal gyrus, PFC: prefrontal cortex, MNI: Montreal Neurological Institute, L: left, R: right.

**Table 3:** Significant group differences between HC and MI while viewing aversive versus positive pictures during the emotional task-based fMRI paradigm.

Group Contrast	Number of Voxels	MNI Coordinate	Region	T Value	*p* Value
HC > MI recovery phase	122	2, 44, −22	R orbitofrontal	3.82	0.000
	55	−26, 28, −18	L orbital part of the IFG	4.21	0.000
	33	−26, 28, −18	L orbitofrontal	4.22	0.000
MI acute phase > MI recovery phase	45	34, −30, −7	R caudate	5.50	0.000
	45	−30, 28, −14	L orbital part of the IFG	4.53	0.000
	29	14, 28, −16	R orbitofrontal	4.78	0.000

IFG: inferior frontal gyrus, MNI: Montreal Neurological Institute, L: left, R: right.

**Table 4:** Significant group differences between TTS and MI while viewing the aversive versus positive pictures during the emotional task-based fMRI paradigm.

Group Contrast	Number of Voxels	MNI Coordinate	Region	T Value	*p* Value
MI acute phase > TTS acute phase	33	52, −52, 16	R angular gyrus	4.06	0.000
	22	42, 24, 22	R dorsolateral PFC	3.97	0.000
	16	−48, 22, 30	L dorsolateral PFC	3.82	0.000
	14	−30, 28, −12	L orbital part of the IFG	4.13	0.000
TTS recovery phase > MI recovery phase	168	−12, 16, −18	L orbitofrontal area	5.06	0.000
	86	40, 14, −30	R temporopolar	5.84	0.000
	51	−32, 28, −14	L orbital part of the IFG	4.03	0.000
	30	−46, 0, 0	L insula	4.07	0.000
	27	42, 0, 8	R insula	4.30	0.000
	13	32, 60, −10	R anterior PFC	4.13	0.000
	9	16, 28, −16	R orbitofrontal area	3.85	0.000
	6	−14, −26, 8	L thalamus	3.76	0.000

IFG: inferior frontal gyrus, PFC: prefrontal cortex, MNI: Montreal Neurological Institute, L: left, R: right.

## Data Availability

The data presented in this study are available on request from the corresponding author.
